# Topographic analysis of K-*ras* mutations in histologically normal lung tissues and tumours of lung cancer patients

**DOI:** 10.1054/bjoc.2001.1913

**Published:** 2001-07

**Authors:** P Keohavong, H H Mady, W M Gao, J M Siegfried, J D Luketich, M F Melhem

**Affiliations:** Departments of Environmental and Occupational Health, Pathology, Pharmacology, Surgery, University of Pittsburgh Cancer Institute, University of Pittsburgh, Pittsburgh, PA, 15213; the Veterans Administration Health Care System, Pittsburgh, PA, 15240, USA

**Keywords:** lung carcinoma, *ras* oncogene, laser capture microdissection, denaturing gradient gel electrophoresis

## Abstract

Mutations in the K-*ras* gene are very common in lung tumours and are implicated in the development of lung cancer, but the timing of their occurrence remains poorly understood. We investigated K-*ras* mutations in cell samples microdissected by laser capture microscopy at multiple sites from lung tissue sections representing tumour tissue and matched histologically normal tissue obtained from 48 lung cancer patients. K-*ras* mutations were detected in cell samples from 10 of 38 (26.3%) lung adenocarcinomas and in none of the histologically normal or tumour cell samples taken from 10 lung squamous cell carcinomas. Of the K-*ras* mutation-positive adenocarcinomas, in 4 cases a mutation was found in only the tumour tissue, in 1 case a mutation was found only in the histologically normal tissue, and in 5 cases mutations were found in both the tumour tissue and histologically normal tissue. Among these 5 cases, 2 had identical mutations in both the tumour tissue and histologically normal tissue, 2 had 1 mutation in the tumour tissue and 2 mutations in the histologically normal tissue, 1 of which was identical to the mutation found in the tumour, and 1 case had 2 codon 12 mutations in tumour tissue and 2 mutations, in codons 9 and 11, in histologically normal tissue. These results showed that K-*ras* mutations are frequent in histologically normal cells taken from outside lung adenocarcinomas and suggest that some of these mutations may represent early events which could pave the way of lung carcinogenesis. © 2001 Cancer Research Campaign http://www.bjcancer.com


*British Journal of Cancer* (2001) **85**(2), 235–241
© 2001 Cancer Research Campaign
doi: 10.1054/ bjoc.2001.1913, available online at http://www.idealibrary.com on IDEAL®

### Topographic analysis of K-*ras* mutations in histologically normal lung tissues and tumours of lung cancer patients

**P Keohavong[1,5], HH Mady[2,6], WM Gao[1], JM Siegfried[3,5], JD Luketich[4,5] and MF Melhem[2,6]**

Departments of [1]Environmental and Occupational Health, [2]Pathology, [3]Pharmacology, and [4]Surgery, and [5]University of Pittsburgh Cancer Institute, University of Pittsburgh, Pittsburgh, PA 15213, and, [6]the Veterans Administration Health Care System, Pittsburgh, PA 15240, USA

**Summary** Mutations in the K-*ras* gene are very common in lung tumours and are implicated in the development of lung cancer, but the timing of their occurrence remains poorly understood. We investigated K-*ras* mutations in cell samples microdissected by laser capture microscopy at multiple sites from lung tissue sections representing tumour tissue and matched histologically normal tissue obtained from 48 lung cancer patients. K-*ras* mutations were detected in cell samples from 10 of 38 (26.3%) lung adenocarcinomas and in none of the histologically normal or tumour cell samples taken from 10 lung squamous cell carcinomas. Of the K-*ras* mutation-positive adenocarcinomas, in 4 cases a mutation was found in only the tumour tissue, in 1 case a mutation was found only in the histologically normal tissue, and in 5 cases mutations were found in both the tumour tissue and histologically normal tissue. Among these 5 cases, 2 had identical mutations in both the tumour tissue and histologically normal tissue, 2 had 1 mutation in the tumour tissue and 2 mutations in the histologically normal tissue, 1 of which was identical to the mutation found in the tumour, and 1 case had 2 codon 12 mutations in tumour tissue and 2 mutations, in codons 9 and 11, in histologically normal tissue. These results showed that K-*ras* mutations are frequent in histologically normal cells taken from outside lung adenocarcinomas and suggest that some of these mutations may represent early events which could pave the way of lung carcinogenesis. © 2001 Cancer Research Campaign http://www.bjcancer.com

**Keywords**: lung carcinoma; *ras* oncogene; laser capture microdissection; denaturing gradient gel electrophoresis

Lung cancer kills both men and women in the United States more than any other type of cancers, causing an estimated 156 900 deaths in 2000 and accounting for 28% of all cancer deaths, in spite of improved therapy available and nationwide anti-smoking campaign (Cancer Facts and Figures – 2000, American Cancer Society). Extensive studies have shown that the development of lung cancer, like many other cancers, is a multistep process which takes several years and proceeds presumably through a series of molecular events leading to an accumulation of genetic variation including mutational, chromosomal and epigenetic changes (Harris, 1991; Sozzi et al, 1995; Stanley, 1995). In this paradigm, one major pathway to lung malignancy involves structural alterations of cancer-related genes, including activation of dominant oncogenes and inactivation of tumour suppressor genes (Barbacid, 1987; Bishop, 1991; Hollstein, 1991; Gazdar, 1994; Stanley, 1995)

The K-*ras* oncogene has been frequently found in mutated form in lung tumours and cell lines; K-*ras* mutations are often associated with the development of lung cancer. This gene codes for a 21-kd protein (*ras*), which is related to the G proteins which bind guanine nucleotides with high affinity and are located at the inner surface of the cell membrane. These proteins have an important role in the signal transduction pathway (Barbacid, 1987; Bishop, 1991; Khosravi-Far and Der, 1994). Activation of the *ras* genes occurs with specific point mutations at only a very few codons including codons 12, 13 or 61, with codon 12 being mutated in more than 90% of K-*ras* mutations identified in lung tumours (Suzuki et al, 1990; Sugio et al, 1992; Rodenhuis and Slebos, 1992; Husgafvel-Pursiainen et al, 1993; Rosell et al, 1996; Keohavong et al, 1996). These mutations induce structural changes within the *ras* proteins leading to an 'activated' GTP-bound conformation.

Lung cancer has been grouped into 4 major histologic types, including adenocarcinoma, squamous cell carcinoma, large cell carcinoma, and small cell lung carcinoma (SCLC). The first 3 types are termed collectively non-small cell lung carcinoma (NSCLC) and have different clinical features from SCLC (Minna et al, 1989). K-*ras* mutations occur in 15% to 50% of adenocarcinoma and large cell undifferentiated carcinoma of the lung. Particularly, they have been more frequently found in tumours from smokers (21–35%) than in those from nonsmokers (5–7%), suggesting their formation may be associated with tobacco smoke exposure. They have also been found, but at a lower frequency, in other forms of pulmonary neoplasia, including squamous cell carcinoma (Suzuki et al, 1990; Sugio et al, 1992; Rodenhuis and Slebos 1992; Husgafvel-Pursiainen, 1993; Rosell et al, 1996; Keohavong et al, 1996, 1997a).

Although K-*ras* mutations have been frequently found in lung tumours, the timing of their occurrence in the multistep process of lung carcinogenesis remains poorly understood. Such studies used tissue specimens removed at biopsy or resection (Sugio et al, 1992; Westra et al, 1993; Clements et al, 1995; Yakubovskaya et al, 1995; Urban et al, 1996; Keohavong et al, 1997b; Urban et al, 2000), and cell samples microdissected from embedded lung tissue sections (Sugio et al, 1994; Westra et al, 1996). Taken

*Received 19 January 2001*
*Revised 23 April 2001*
*Accepted 30 April 2001*

*Correspondence to:* P Keohavong together, some of these studies suggest that K-*ras* mutations occur relatively late in lung cancer development (Sugio et al, 1994; Yakubovskaya et al, 1995; Urban et al, 1996), while other studies indicate that they could represent early events in this process (Li et al, 1994; Clements et al, 1995; Keohavong et al, 1997b; Urban et al, 2000).

We have previously applied a sensitive method to analyse and compare K-*ras* mutations in lung tumour and matched tumour-adjacent histologically normal tissues (Keohavong et al, 1997). We have reported that histologically normal tissues taken from outside K-*ras*-mutated lung tumours contained a low fraction of the mutational genotype identical to those found in the corresponding tumour in 4 of 8 lung cancer patients. However, the fact that such a study used tissue samples that each contained several hundreds cells, there were some concerns that the low fraction mutations detected in the histologically normal tissues might have originated from a contamination with very few tumour cells or mutated DNA diffusing from necrotic tumour cells in surrounding tissues, although we took all precautions to avoid or at least minimize such a possibility (Keohavong et al, 1997b). In this study, we applied a laser capture microdissection technique to histopathologically review and precisely sample, under direct microscope visualization, a few cells from various areas of paraffin-embedded lung tissue sections, including histologically normal tissue and matched tumour tissue, obtained from 48 cases of lung cancer patients. We analysed K-*ras* mutations in each cell sample using polymerase chain reaction and denaturing gradient gel electrophoresis. The results demonstrate that K-*ras* mutations are present in histologically normal tissue surrounding lung tumour in about 15.8% (6 of 38) of patients with lung adenocarcinoma. We compared these results with those reported previously by us and others and discussed them in relation to the role of K-*ras* mutations in lung carcinogenesis and as potential surrogate biomarkers for lung cancer.

### MATERIALS AND METHODS

### Patients and tissue specimens

Pulmonary lobectomy specimens were obtained from 48 patients with lung cancer, including 38 adenocarcinoma and 10 squamous cell carcinoma. The demographic and clinical information is shown in Table 1. These specimens were formalin-fixed and paraffin- embedded and stored at the University of Pittsburgh Medical Center between 1990 and 1999. The diagnosis of lung adenocarcinoma and squamous cell carcinoma was confirmed, using established morphologic criteria. For DNA analysis, 5 μm histologic sections were prepared from multiple paraffin blocks representative of tumour and those representative of matched histologically normal lung tissues (located between 2.0 cm and 5.0 cm from the tumour edge). These normal tissues corresponded to either bronchial or bronchiolar pseudostratified columnar epithelial tissues that matched tumours (adenocarcinoma or squamous cell carcinoma) of a bronchial origin, or alveolar walls that matched tumors (adenocarcinoma or bronchoalveolar type) of an alveolar origin. All tissue sections were individually deparaffinized in xylene and then in ethanol, rinsed with water, and air-dried. All slides were stained with hematoxylin and eosin and reviewed to confirm the presence of histologically normal tissue and/or tumour. The microdissection was performed by a trained pathologist using a laser capture microdissection microscope (PixCell II LCM System, Arcturus Engineering, Mountain View, CA). With the aid of this new technique, individual cells within areas of interest of a tissue section were isolated (Emmert-Buck et al, 1996). Areas of tumour tissue and histologically normal tissue were prepared and microdissected separately to avoid any possible cross-contamination between tissues. Samples of 100–200 histologically normal cells or malignant cells were 'laser-captured' on separate 'Caps' built to fit a 0.5 ml microcentrifuge tube (Acturus Engineering, Mountain View, CA). Each cell sample was considered to be 100% 'homogeneously' normal-appearing or malignant, as determined by a careful microscopic visualization of the cells captured on each Cap.

### DNA extraction and K-*ras* codon 12 mutation analysis

The captured cells were lysed by adding directly on each Cap 20 μl of lysis solution (40 mM Tris-HCl, pH 8.0, 1 mM EDTA, 0.5% Tween-20, and 0.5 μg $\mu l^{-1}$ proteinase K) (Sugio et al, 1994). The Cap was enclosed into a 0.5 ml microcentrifuge tube (in an upside-down position) and incubated at room temperature for 48 hours with occasional gentle shaking. The resulting cell lysate was recovered in the tube by a quick and gentle spinning in a microcentrifuge then heated at 95°C for 5 minutes to inactivate the proteinase K. Half (10 μl) of each cell lysate was used as template for a first round of PCR to amplify a 75-bp genomic fragment containing the 5′ half of exon 1 of the K-*ras* gene and adjacent flanking intron sequence. PCR was carried out in a final 50 μl reaction mixture containing 10 mM Tris-HCl, pH 8.3, 1.5 mM $MgCl_2$, 50 mM KCl, 100 μM each dNTP, 0.5 μM each primer and 2.5 units of Gold AmpliTaq DNA polymerase (Perkin Elmer, CT). The mixture was heated at 95°C for 9 minutes then subjected to 15 cycles (94°C/1 minute, 53°C/1 minute, 72°C/1 minute), using the primers KI1-1 (sense): 5′- TATTATAAGGCCTGCTGAAA-3′ and PKB (antisense): 5′- AGGCACTCTTGCCTACGGCA-3′. For the second round of PCR, 2 μl of the PCR products from the first round were diluted into a final 25 μl reaction mixture containing the same buffer composition as above except that 0.25 μl of [α-$^{32}$P]-dATP (3000 Ci/mmol, New England Nuclear, Boston, MA) was added and primer KI1-1 was replaced by primer PKGC: 5′-GCCGCCTGCAGCCCGCGCCCCCCGTGCCCCCGCCCCGC-CGCCGGCCCGGCGCCTATAAGGCCTGCTGAAAATG-3′. The mixture was heated at 95°C for 9 minutes then subjected to 35 PCR cycles (94°C/1 minute, 60°C/1 minute, 72°C/1 minute). The resulting PCR products were separated by gel electrophoresis and autoradiographed. Band containing the expected-size 126-bp fragment was excised from the gel. DNA was eluted from the gel slice and analysed by DGGE (Bio-Rad, CA) under the conditions described previously (Keohavong et al, 1997b). Mutant alleles were isolated from the denaturing gradient gel and further characterized by sequencing. This method allows us to clearly detect a K-*ras* mutant allele present at a mutant fraction of 5% (1 mutant allele among 20 wild-type alleles) (Keohavong et al, 1997b). This level of sensitivity should be sufficient to identify K-*ras* mutations in DNA samples from an equivalent 50–100 original cells. In the cases where mutant alleles appeared ambiguous by using the above PCR+DGGE method, a more sensitive version of this method which has a detection limit of $10^{-3}$–$10^{-4}$ (one mutant allele in $10^3$–$10^4$ wild-type allele) was used. This method requires an additional step for mutant allele enrichment involving a prior treatment of the 126-bp amplified fragment with Ban I restriction enzyme which cleaves fragments with a non-mutated K-*ras* codon

© 2001 Cancer Research Campaign

12. The digestion-resistant fragment was gel-purified and analysed by DGGE as described previously (Keohavong et al, 1997b).

### RESULTS

### Lung tissue sampling and microdissection

Figure 1 shows a representative example of a target-site sampling from a case of lung adenocarcinoma. The use of a laser capture microscopy allowed an accurate morphologic analysis and precise sampling of cells from areas of interest in paraffin-embedded tissue sections. Histologically normal cells were individually resected by laser capture from the tissue area located between 2 cm and 5 cm from the edge of the tumour area. Between 100 and 200 cells were captured on each Cap after a careful histologic examination and determination of their benign morphology. All cells appeared homogeneously normal-looking and there were no tumour cells present. Paraffin-embedded tissue blocks representative of tumour tissue were topographically sampled in a similar manner. The cells captured from the tumour tissue also all appeared malignant histologically. After microdissection, each captured cell sample was compared to the matched tissue section prior to and after microdissection to confirm the accuracy of target selection (see Figure 1A–D). Using this approach, we prepared 38 cell samples from histologically normal tissue and 38 cell samples from matched malignant tissue from paraffin-embedded lung tissue sections selected from 38 patients with lung adenocarcinoma. We also prepared cell samples from histologically normal tissue, matched metaplasia and tumour tissue from paraffin-embedded tissue sections obtained from 10 patients with lung squamous cell carcinoma.

### Mutation analysis

Molecular analysis of the above cell samples using the PCR+DGGE method yielded 10 lung cancer cases showing K-*ras* mutant alleles in tumour cells and/or paired histologically normal cells, all of which corresponded to lung adenocarcinoma (26.3% or 10 of 38 cases). Figure 2 shows an example of DGGE separation of K-*ras* mutant alleles from the wild-type allele for 6 of the 10 K-*ras* mutant-positive cases. The mutant alleles were isolated from the gel and the nature of the mutations was determined by

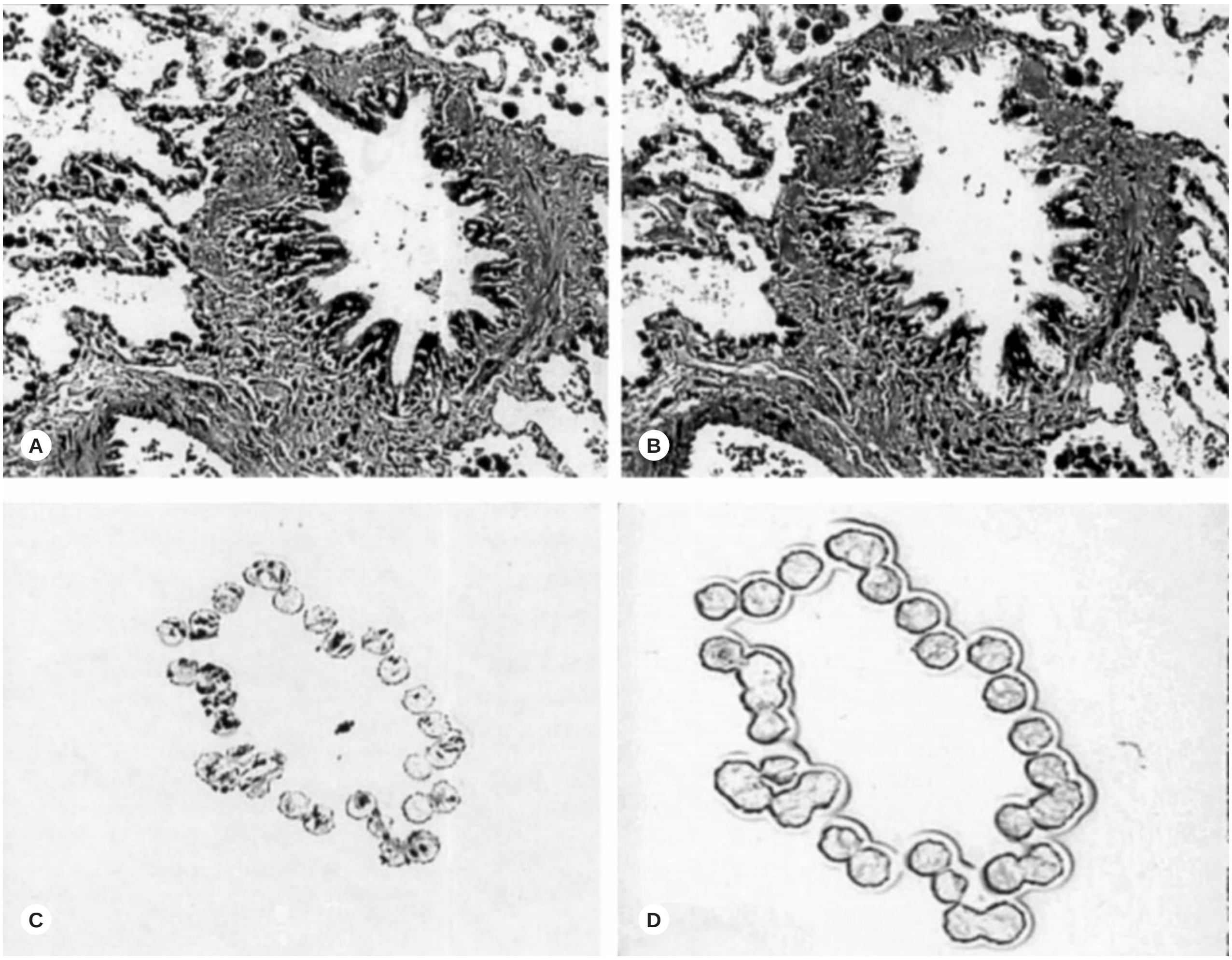

**Figure 1** An example of tissue site sampling from a formalin-fixed and paraffin-embedded tissue section from one case of lung adenocarcinoma (see patient 4 in Table 1) by using a laser capture microdissection microscope (magnification × 100). The figure shows a tissue section obtained from a block representative of a histologically normal lung tissue pre- (**A**) and post- (**B**) topographic microdissection of normal-appearing cells from an area of interest of the tissue section. The cells were ‘captured’ on a Cap (**C**) and reviewed further for cell population homogeneity and then treated with proteinase K. (**D**) shows the same cell sample after treatment with proteinase K, with the cellular content being totally lysed. Under the lysis conditions shown in Materials and Methods, between 95% and 100% of the captured cells were completely lysed

© 2001 Cancer Research Campaign 

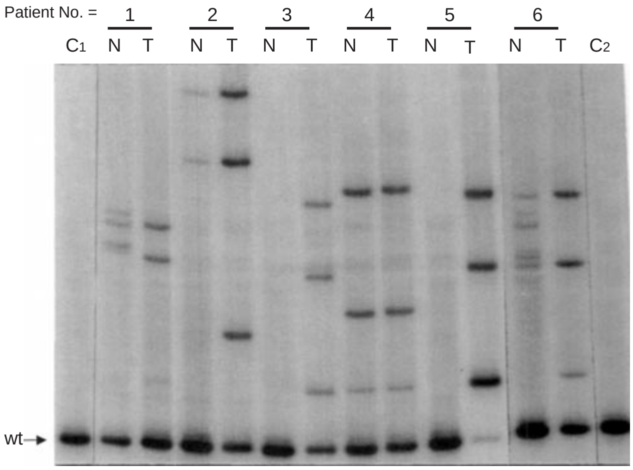

**Figure 2** DGGE analysis of K-*ras* mutations, K-*ras* gene exon 1 is amplified from cellular DNA isolated from each cell sample microdissected from histologically normal (N) and tumour (T) areas of a selected lung tissue section as shown in Figure 1. The amplified DNA is analysed by DGGE to separate any K-*ras* mutant allele that may be present in each cell sample from the wild type allele. The figure shows sequence patterns of K-*ras* mutant alleles for 6 of 10 lung adenocarcinoma that were found to be positive for K-*ras* mutation in the tumour tissue and/or the histologically normal tissue. The wild-type allele was present in excess over mutant allele(s) in each DNA sample, except for the tumour DNA sample in patient 5, and was detected as a homoduplex fragment (wt). The presence of a mutant allele was detected as 3 additional fragments, including a mutant homoduplex and the 2 respective mutant/wild-type heteroduplexes that separated from the wild-type fragment in lower denaturant concentrations from the bottom to the top of the gel, respectively. In patient 1, the histologically normal DNA sample showed 4 mutant/wild-type heteroduplexes that corresponded to the presence of 2 different mutant alleles (see patient 1 in Table 1). Lanes C show the sequence patterns of negative control DNA samples corresponding to a known K-*ras* mutation-negative benign lung tissue (C1) and infant normal intestine tissue (C2). The histologically normal tissue sample (N) in patient 6 contained several faint heteroduplex bands. Further experiments showed that only the 2 heteroduplexes identical to those appearing in the matched tumour sample (T) were repeatedly detected. Therefore, the presence of the other heteroduplexes in the histologically normal tissue sample (N) was presumably due to experimental variation sequencing. No mutants were identified in any of the cell samples corresponding to tumour tissue or paired histologically normal tissue obtained from the 10 cases of squamous cell carcinoma (data not shown). These results are in line with previous studies showing that K-*ras* mutations were frequently identified in lung adenocarcinoma but were rarely so in lung squamous cell carcinoma (Rodenhuis and Slebos, 1992; Husgafvel-Pursiainen et al, 1993; Keohavong et al, 1996). As shown in Figure 2, the 2 control samples of known normal tissues (C1 and C2) revealed only the K-*ras* codon 12 wild-type homoduplex (wt). Compared with the controls, patients 3 and 5 showed each a mutant pattern in the tumour tissue but not in the paired histologically normal tissue. These patients harboured each a GGT to TGT mutation in their lung tumour tissue. In addition, in patient 5, the wild-type allele appeared only as a small fraction of the mutant allele in this tumour sample (T). This observation may indicate that some tumour cell in this sample may have lost 1 allele of K-*ras* gene while containing a mutation in the remaining allele. This loss of the wild-type allele appeared to occur at a specific area of the tumour tissue because further analysis of tumour cells taken from 2 separate sites of tumour tissue from this patient showed not only the TGT mutation but also the wild-type allele at a fraction higher than the TGT mutant allele in both samples (data not shown). Nevertheless, the presence of a loss of 1 allele and a mutation in the other allele of the K-*ras* gene has not been reported previously. It may be that the deletion of the wild-type allele occurred as a secondary event that accumulated during the progression of cells with already a K-*ras* mutated gene in one allele into tumour. Patients 2, 4 and 6 each harboured a genotypically identical mutation in both the tumour tissue and the paired histologically normal tissue, corresponding to a GGT to TTT, a GGT to GTT, and GGT to TGT, respectively. Patient 1 showed 4 mutant/wild-type heteroduplexes in the histologically normal tissue (N), which were found to correspond to 2 different mutations, a GTT to ATT in codon 9, and a GCT to ACT in codon 11, of the K-*ras* gene. This patient also showed two mutant/wild-type heteroduplexes in the matched tumour tissue (T), which corresponded to a GGT to GAT mutation in codon 12 of the K-*ras* gene.

### Multiple site analysis of K-*ras* mutations

In order to investigate in more detail the topographic distribution of mutations in each lung tumour tissue and histologically normal tissue, all 10 K-*ras* mutation-positive adenocarcinomas as well as 3 K-*ras* mutation-negative adenocarcinomas were re-examined further for K-*ras* mutations. Toward this end, between 3 and 5 additional successive tissue sections were prepared from original tissue blocks representing tumour-surrounding histologically normal tissue as well as those representing tumour tissue for each cancer case. Table 1 summarizes the final results of K-*ras* mutations identified at multiple sites in both the histologically normal tissue and the matched tumour tissue for the 10 cases of K-*ras* mutation-positive lung adenocarcinomas. The cell samples obtained from the tumour tissue and histologically normal tissue for the 3 selected K-*ras* mutation-negative patients were all found to be negative for K-*ras* mutations (data not shown), confirming the results of the first experiment. 4 of the 10 K-*ras* mutation-positive cases (Patients 3, 5, 7 and 8) contained each a mutation (a GGT to TGT) in only the tumour tissue. This mutation was present in all malignant cell samples taken from 3 different areas of the tumour tissue from each of these patients. Patients 2 and 6 each contained a genotypically identical mutation (a GGT to TTT and GGT to TGT, respectively) in both the tumour tissue and matched histologically normal tissue. These mutations were present in all cell samples taken from 3 different tissue blocks representative of the normal-appearing tissue and those representative of the matched tumour tissue obtained from these patients, in agreement with the results of the first experiment shown in Figure 2 for these patients. Likewise, multiple site analysis helped confirm that Patient 4 contained a genotypically identical mutation, a GGT to GTT, in both the tumour tissue and histologically normal tissue. In addition, as shown in Figure 3, a second mutation, a GGT to AGT, was detected in the histologically normal tissue. However, while the GGT to GTT mutation was found in all tissue blocks representative of the histologically tissue (see lanes N1, N2 and N3 and n1 n2, and n3), as well as those representative of the tumour tissue (see T1, T2 and T3), the GGT to AGT mutation was found in only cell samples taken from one specific area of the histologically normal tissue (see lanes N1 and n1). This tissue area was defined at the margin of resection located 5 cm from the edge of the tumour. In the remaining 3 patients (Patients 1, 9 and 10; Table 1), the mutations were identified at a low fraction as shown by the fact they were identified only in 1 or 2 of the total 6 cell samples taken from the histologically normal tissue and/or the tumour tissue and by only using a more sensitive version of the

 © 2001 Cancer Research Campaign

**Table 1** Summary of K-*ras* mutations in lung tissue sections from 10 cases of lung adenocarcinoma[a]

| Patient no. | Gender | Age | Mutations | | |
|---|---|---|---|---|---|
| | | | **Normal tissue** | **Tumour tissue** | **Amino acid change** |
| 1 | M | 62 | cod.9 (GTT to ATT) | | Val to Ile |
| | | | cod.11 (GCT to ACT) | | Ala to Thr |
| | | | | GAT | Asp |
| | | | | AGT | Ser |
| 2 | F | 48 | TTT | TTT | Phe |
| 3 | M | 66 | none | TGT | Cys |
| 4 | M | 53 | GTT | GTT | Val |
| | | | AGT | | Ser |
| 5 | M | 60 | none | TGT | Cys |
| 6 | M | 74 | TGT | TGT | Cys |
| 7 | M | 53 | none | TGT | Cys |
| 8 | M | 72 | none | TGT | Cys |
| 9 | M | 66 | GAT | | Asp |
| | | | AGT | AGT | Ser |
| 10 | F | 72 | AGT | none | Ser |

[a]Except for the 2 mutations found in histologically normal tissues in patient 1, all mutations shown involved codon 12 of the K-*ras* gene.

PCR + DGGE method (see Materials and Methods). Patient 1 contained 2 mutations in codon 12 (a GGT to GAT and GGT to AGT) in tumour tissue and 2 mutations, 1 in codon 9 (a GTT to ATT) and 1 in codon 11 (a GCT to ACT), in histologically normal tissue. These mutations were identified each in only 1 or 2 of the 6 samples taken from either the tumour tissue or the histologically normal tissue. In Patient 9, 2 mutations (a GGT to GAT and GGT to AGT) were found in histologically normal tissue and 1 mutation (a GGT to AGT) was found in tumour tissue. In Patient 10, 1 mutation (a GGT to AGT) was found in 2 of 6 samples taken from the histologically normal tissue and in none of 6 malignant cell samples.

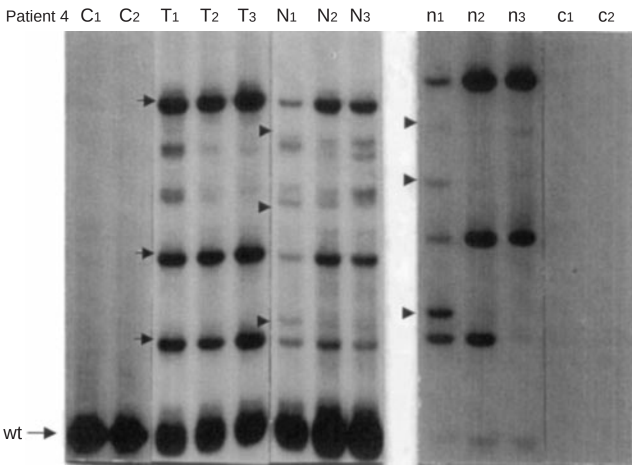

**Figure 3** An example of DGGE analysis of K-*ras* mutations at multiple sites of lung tissue sections for patient 4 shown in Table 1. The figure shows the GGT to GTT mutant sequence pattern in cells sampled at 3 regions of the tumour tissue (T1, T2, T3) and 3 regions of the histologically normal tissue (N1, N2, N3) corresponding to patient 4. The histologically normal cells were sampled at 5 cm (N1 and N2) and 2 cm (N3) from the edge of the tumour. Lanes C1 and C2 contained DNA with non-mutated K-*ras* gene corresponding to benign lung tissue and infant intestinal tissue, respectively. Shown in lanes n1, n2, n3, and c1 and c2, are the same DNA from N1, N2, N3, and C1 and C2, that had been first subjected to BanI restriction enzyme digestion to eliminate any non-K-*ras* codon 12 mutant allele, including the wild-type allele (wt), and thereby to enrich for K-*ras* codon 12 mutant alleles, then analysed by DGGE (Keohavong et al, 1997). The positions of the mutant homoduplexes (second fragment from wt) and the 2 respective mutant/wild-type heteroduplexes (third and fourth fragments from wt) were indicated by arrows for the GGT to GTT mutation in lanes T1–T3, and by arrowheads for the GGT to AGT mutation in lanes N1–N3 and n1. After mutant allele-enrichment by BanI digestion, the GGT to AGT mutant pattern in lane N1 appeared more clearly visible in lane n1. As expected, control DNA shown in lanes c1 and c2 did not contain any K-*ras* codon 12 mutant allele

### DISCUSSION

Although K-*ras* mutations have been frequently found in lung tumours and tumour cell lines and implicated in the development of lung cancer, the timing of these mutations in lung carcinogenesis remains poorly understood. There have been several but conflicting reports on whether or not K-*ras* mutations are present in non-neoplastic or normal-appearing lung tissues, which, in the affirmative, would indicate that they occur early during lung cancer development and can provide an early lung cancer detection marker.

We applied a laser capture microdissection microscope to morphologically review and sample cells at multiple sites from both lung tumour tissue and histologically normal tissue adjacent to as well as those distant from the tumour. Our results, based on independent experiments using successive lung tissue sections from multiple tissue blocks, demonstrated that K-*ras* mutations were present in histologically normal tissue in 6 of 10 (60%) K-*ras* mutation-positive lung adenocarcinomas, or 6 of all 38 (15.8%) lung adenocarcinomas examined. Therefore, K-*ras* mutations are relatively frequent in histologically normal lung tissue surrounding malignant tissue in lung adenocarcinomas. These mutations can be grouped into high fraction mutations and low fraction mutations in tumour and/or histologically normal tissue. Low fraction K-*ras* mutations have been previously reported in lung tumour DNA by us and others (Mills et al, 1995; Keohavong et al, 1997a). They were frequently identified in tumour tissue as well as in histologically normal tissue and metastasis (Yakubovskaya et al, 1995). Our present study showed that mutations in patients 1, 9 and 10 were detected in only some cell samples taken from malignant tissue and/or matched histologically normal tissue, suggesting that they were not initiating or early events in lung carcinogenesis but rather secondary events accumulating in

*© 2001 Cancer Research Campaign* 

subclones during tumour progression or as a result of continued exposure to tobacco smoke (Mills et al, 1995; Yakubovskaya et al, 1995; Keohavong et al, 1997a). The GTT to ATT mutation (codon 9) and GCT to ACT mutation (codon 11) found in patient 1 had not been reported previously and their biological significance in lung carcinogenesis is not known. Among the lung adenocarcinomas with high fraction mutations, 2 observations can be made. First, these mutations can be found in only the tumour tissue and not in the matched histologically normal tissue (Patients 3, 5, 7 and 8). The fact that they were detected at multiple sites in lung tumour tissue suggests that they may represent events occurring early in small precursor lesions before tumour progression. Secondly, some K-*ras* mutations are found not only in the tumour tissue but also, at a mutant fraction generally lower than that detected in the tumour tissue, in histologically normal tissues adjacent to as well as those distant from the tumour (Patients 2, 4 and 6). These results confirmed our previous study showing that genotypically identical K-*ras* mutations were detected in both the tumour tissue and, at a lower mutant fraction, in tumour-adjacent normal-appearing tissue in 4 of 8 cases of lung adenocarcinoma (Keohavong et al, 1997). These mutations may represent early events, perhaps initiating events, in the development of these tumours. Furthermore, Patient 4 provides an intriguing example since only 1 mutation, a GGT to GTT, is present in the tumour tissue, but 2 mutations, a GGT to GTT and GGT to AGT, are present in the matched histologically normal tissue. In addition, while the GTT mutation was detected in all samples taken from 6 different sites of the histologically normal tissue, the GGT to AGT mutation was detected in only cell samples taken from a defined area of the normal-appearing tissue distant from the tumour. This observation is compatible with the existence of 2 overlapping fields with each a different mutated K-*ras* gene, and the field with the GTT mutation may contribute to the development of this tumour (Slaughter et al, 1953; Strong et al, 1984). It is, however, unclear why a field with a specific mutation would have a higher ability to develop into a tumour than one with another mutation type. Some studies suggested that the substitution of the wild-type codon 12 amino acid (glycine) with certain amino acids, including valine and arginine, in the H-*ras* gene may give rise to gene products with a more potent transforming ability than the wild-type or other amino acids (Seeburgh et al, 1984; Der et al, 1986). It will require an expanded study involving a larger number of lung cancer patients to determine whether our explanation for the observation in patient 4 can be supported.

K-*ras* mutations were detected in histologically normal mucosal tissues adjacent to or distant from colorectal carcinoma (Pretlow et al, 1991; Minamoto et al, 1995; Zhu et al, 1997). They have been also suggested to be preneoplastic events in pancreatic cancer (Yanagisawa et al, 1993; Berthelemy et al, 1995; Brentall et al, 1995; Tada et al, 1996; Wilentz et al, 1998). However, in lung cancer, there have been conflicting reports on K-*ras* mutations in non-neoplastic tissues. On the one hand, Santos et al (1984) investigated K-*ras* codon 12 mutation in lung tissues obtained from a patient with squamous cell carcinoma of the lung and reported that the mutation detected in lung carcinoma was absent from the normal bronchial and parenchymal tissues of that patient. The authors suggested that malignant activation of K-*ras* oncogene may be specifically associated with the development of a human neoplasm. Sugio et al (1994) reported that K-*ras* mutations were absent in metaplasia and normal-appearing cells of the lung and were infrequent in dysplastic lesions, suggesting that these mutations are relatively late events in the pathogenesis of lung cancer. Likewise, Urban et al (1996) did not detect any K-*ras* mutation in non-neoplastic peripheral bronchial or parenchymal tissues associated with lung tumours. Finally, Yakubovskaya et al (1995) detected K-*ras* mutations in 60% of normal-appearing lung tissue samples, 63% of tissue samples from tumour tissue, and 80% of samples of metastasis obtained from patients with non-small cell lung cancer. However, the fact these mutations were present at a low fraction suggests that they did not contribute to the development of lung cancer. On the other hand, the results of these studies are not in agreement with other published studies. For instance, K-*ras* mutations were identified in atypical alveolar hyperplasia (AAH), a potential precursor from which lung adenocarcinoma arises (Westra et al, 1996). In another study, Clements et al (1995) analysed bronchoscopy specimens obtained from lung cancer patients and showed that K-*ras* mutations were found in both malignant and nonmalignant tissues in 22.7% (5 of 22) of the patients and in only nonmalignant tissue in 9.1% (2 of 22) of the patients. Finally, a recent study by Urban et al (2000) showed that K-*ras* mutations were detected in both lung tumour and bronchial carina tissue in 21% (4 of 19) of the patients, while being present only in the bronchial carina but not in the tumour in 11% (2 of 19) of the patients. These latter studies and our present study showed that K-*ras* mutations can be frequently found in non-neoplastic lung tissues, including histologically normal tissues.

Questions remain about the usefulness of K-*ras* mutations as an early detection markers for lung cancer. Mutation in the K-*ras* gene is a well-established example of the association between environmental exposure (chiefly tobacco smoke) and human lung cancer, since mutations in the K-*ras* gene are found almost exclusively in lung tumours from smokers, while being rare in lung tumours from non-smokers (Rodenhius and Slebos, 1992; Sugio et al, 1992; Suzuki et al, 1990; Husgafvel-Pursianen et al, 1993; Keohavong et al, 1996; Rosell et al, 1996). In theory, these mutations should be useful markers of exposure to carcinogens in tobacco smoke, and their detection in high-risk individuals without cancer would be an indication of damaged airway epithelium. The presence of these mutations in airways of ex-smokers might also be an indication of persistence of genetic damage in ‘condemned epithelium’. The technique of laser capture microdissection as described here could be applied to mucosal biopsies taken by either white light or fluorescent bronchoscopy to determine if K-*ras* mutations are present in preneoplastic or histologically normal epithelium from the airways of individuals at high risk for lung cancer.

### ACKNOWLEDGEMENTS

This work was supported by a grant from the American Cancer Society (RPG-99-161-01-CNE), and by an NCI grant 1-RO3-CA71609-01, and The Veterans Research Foundation of Pittsburgh, Pittsburgh, PA15240.

### REFERENCES

American Cancer Society (2000) Cancer Facts and Figures – 2000. *American Cancer Society*

Barbacid M (1987) *ras* genes. *Annu Rev Biochem* **56**: 779–827

Berthelemy P, Bouisson M, Escourrou J, Vaysse N, Rumeau JL and Pradayrol L (1995) Identification of K-*ras* mutations in pancreatic juice in the early diagnosis of pancreatic cancer. *Ann Intern Med* **123**: 188–191

© 2001 Cancer Research Campaign

Bishop JM (1991) Molecular themes in oncogenes. *Cell* **64**: 235–248

Brentnall TA, Chen R, Lee JG, Kimmey MB, Bronner MP, Haggitt RC, Kowdley KV, Hecker LM and Byrd DR (1995) Microsatellite instability and K-*ras* mutations associated with pancreatic adenocarcinoma and pancreatitis. *Cancer Res* **55**: 4264–4267

Clements NC, Nelson MA, Wymer JA, Savage C, Aguirre M and Garewal H (1995) Analysis of K-*ras* gene mutations in malignant and nonmalignant endobronchial tissue obtained by fiberoptic bronchoscopy. *Am J Respir Crit Care Med* **152**: 1374–1378

Der CJ, Finkel T and Cooper GM (1986) Biological and Biochemical properties of human *ras*$^{H}$ genes mutated at codon 61. *Cell* **44**: 167–176

Emmert-Buck MR, Bonner RF, Smith PD, Chuaqui RF, Zhuang Z, Goldstein SR, Weiss RA and Liotta LA (1996) Laser capture microdissection. *Science* **274**: 998–1001

Gazdar AF (1994) The molecular basis of human lung cancer. *Anticancer Res* **13**: 261–268

Harris CC (1991) Chemical and physical carcinogenesis: advances and perspectives. *Cancer Res* **51**: 5023s–5044s

Hollstein M, Sidransky D, Vogelstein B and Harris CC (1991) p53 mutations in human cancers. *Science* **253**: 49–53

Husgafvel-Pursiainen K, Hackman P, Ridanpaa M, Anttila S, Karjalainen A, Partanen T, Taikina-Aho O, Heikkila L and Vaino H (1993) K-*ras* mutations in human adenocarcinoma of the lung: association with smoking and exposure to asbestos. *Int J Cancer* **53**: 250–256

Keohavong P, DeMichele MAA, Melacrinos AC, Landreneau RJ, Weyant RJ and Siegfried JM (1996) Detection of K-*ras* mutations in lung carcinomas: Relationship to prognosis. *Clin Cancer Res* **2**: 411–418

Keohavong P, Zhu D, Melacrinos AC, DeMichele MAA, Weyant RJ, Luketich JD, Testa JR, Fedder M and Siegfried JM (1997a) Detection of low-fraction K-*ras* mutations in primary lung tumors using sensitive methods. *Int J Cancer* **74**: 162–170

Keohavong P, Zhu D, Whiteside TL, Swalsky P, Bakker A, Elder EM, Siegfried JM, Srivastava S and Finkelstein SD (1997b) Detection of infrequent and multiple K-*ras* mutations in human tumors and tumor-adjacent tissues. *Anal Biochem* **247**: 394–403

Khosravi-Far R and Der CJ (1994) The ras signal transduction pathway. *Cancer Metast Rev* **13**: 67–89

Li ZH, Zheng J, Weiss LM and Shibata D (1994) c-K-*ras* and p53 mutations occur very early in adenocarcinoma of the lung. *Am J Pathol* **144**: 303–309

Marshall CJ (1991) Tumor suppressor genes. *Cell* **64**: 313–326

Mills NE, Fishman CL, Rom WN, Dubin N and Jacobson DR (1995) Increased prevalence of K-*ras* oncogene mutations in lung adenocarcinoma. *Cancer Res* **55**: 1444–1447

Minamoto T, Yamashita N, Ochiai A, Mai M, Sugimura T, Ronai Z and Esumi H (1995) Mutant K-*ras* in apparently normal mucosa of colorectal cancer patients: its potential as a biomarker of colorectal tumorigenesis. *Cancer* **75**: 1520–1526

Minna JD, Pass H, Glatstein E and Ihde DC (1989) Lung cancer. In: *Principles and Practice of Oncology*. deVita VT, Rosenberg S and Hellman S (eds). pp. 591–705. Lippincott JB: Philadelphia

Pretlow TP, Barrow BJ, Ashton WS, O'Riordan MA, Pretlow TG, Jurcisek JA and Stellato TA (1991) Aberrant crypts: putative preneoplastic foci in human colonic mucosa. *Cancer Res* **51**: 1564–1567

Rodenhuis S and Slebos RJ (1992) Clinical significance of *ras* oncogene activation in human lung cancer. *Cancer Res* **52**: 2665s–2669s

Rosell R, Monzo M, Pifarre A, Ariza A, Sanchez JJ, Moreno I, Maurel J, Lopez M, Abad A and de Anta JM (1996) Molecular staging of non-small cell lung cancer according to K-*ras* genotypes. *Clin Cancer Res* **2**: 1083–1086

Santos E, Martin-Zanca D, Reddy EP, Pierotti MA, Della Porta G and Barbacid M (1984) Malignant activation of a K-*ras* oncogene in lung carcinoma but not in normal tissue of the same patient. *Science* **223**: 661–664

Seeburgh PH, Colby WW, Capon DJ, Goeddel DV and Levinson AD (1984) Biological properties of human c-H-*ras* genes mutated at codon 12. *Nature* (Lond.) **312**: 71–75

Slaughter TP, Southwick HW and Smejkel W (1953) Field cancerization in oral stratified epithelium. *Cancer* **6**: 963–968

Sozzi G, Miozzo M, Pastorino U, Pilotti S, Donghi R, Giarola M, Gregorio LD, Manenti G, Radice P, Minoletti F, Porta GD and Pierotti MA (1995) Genetic evidence for an independent origin of multiple preneoplastic and neoplastic lung lesion. *Cancer Res* **55**: 135–140

Stanley LA (1995) Molecular aspects of chemical carcinogenesis: the roles of oncogenes and tumor suppressor genes. *Toxicology* **96**: 173–194

Strong MS, Incze J and Vaughan CW (1984) Field cancerization in the aerodigestive tract – its etiology, manifestation, and significance. *J Ontolaryn* **13**: 1–6

Sugio K, Ishida T, Yokoyama H, Inoue T, Sugimachi K and Sasazuki T (1992) *ras* gene mutation as a prognostic marker in adenocarcinoma of the human lung without lymph node metastasis. *Cancer Res* **52**: 2903–2906

Sugio K, Kishimoto Y, Virmani AK, Hung JY and Gazdar AF (1994) K-*ras* mutations are relatively late event in the pathogenesis of lung carcinomas. *Cancer Res* **54**: 5811–5815

Suzuki Y, Orita M, Shiraishi M, Hayashi K and Sekiya T (1990) Detection of *ras* gene mutations in human lung cancer by single-stranded conformational polymorphism analysis of polymerase chain reaction products. *Oncogene* **5**: 1037–1043

Tada M, Ohashi M, Shiratori Y, Okudaira T, Komatsu Y, Kawabe T, Yoshida H, Machinami R, Kishi K and Omata M (1996) Analysis of K-*ras* gene mutation in hyperplastic duct cells of the pancreas without pancreatic disease. *Gastroenterology* **110**: 227–231

Urban T, Ricci S, Lacave R, Antoine M, Kambouchner M, Capron F and Bernaudin JF (1996) Codon 12 Ki-*ras* mutation in non-small-cell lung cancer: comparative evaluation in tumoural and non-tumoural lung. *Brit J Cancer* **74**: 1051–1055

Urban T, Ricci S, Danel C, Antoine M, Kambouchner M, Godard V, Lacave R and Bernaudin JF (2000) Detection of codon 12 K-*ras* mutations in non-neoplastic mucosa from bronchial carina in patients with lung cancer. *Brit J cancer* **82**: 412–417

Westra WH, Slebos RJ, Offerhaus GJ, Goodman SN, Evers SG, Kensler TW, Askin FB, Rodenhius S and Hruban RH (1993) K-*ras* oncogene activation in lung adenocarcinomas from former smokers. *Cancer* **72**: 432–438

Westra WH, Baas IO, Hruban RH, Askin FB, Wilson K, Offenhaus GJA and Slebos RJC (1996) K-*ras* oncogene activation in atypical alveolar hyperplasia of the human lung. *Cancer Res* **56**: 2224–2228

Wilentz RE, Chung CH, Sturm PD, Musler A, Sohn TA, Offerhaus GJ, Yeo CJ, Hruban RH and Slebos RJ (1998) K-*ras* mutations in the duodenal fluid of patients with pancreatic carcinoma. *Cancer* **82**: 96–103

Yakubovskaya MS, Spiegelman V, Luo FC, Malaev F, Salnew A, Zborovskaya I, Gasparian A, Polotsky B, Machaladze Z, Trachtenberg AC, Belitsky GA and Ronai Z (1995) High frequency of K-*ras* mutations in normal appearing lung tissues and sputum of patients with lung cancer. *Int J Cancer* **63**: 810–814

Yanagisawa A, Ohtake K, Ohashi K, Hori M, Kitagawa T, Sugano H and Kato Y (1993) Frequent c-Ki-*ras* oncogene activation in mucous cell hyperplasias of pancreas suffering from chronic inflammation. *Cancer Res* **53**: 953–956

Zhu D, Keohavong P, Finkelstein SD, Swalska P, Bakker A, Weissfeld J, Srivastava S and Whiteside TL (1997) K-*ras* mutations in normal colorectal tissues from K-*ras* mutation-positive colorectal cancer patients. *Cancer Res* **57**: 2485–2492

*© 2001 Cancer Research Campaign*

## References

[PDF_00582] Barbacid M. (1987). ras genes.. Annu Rev Biochem.

[PDF_00583] Berthélemy P., Bouisson M., Escourrou J., Vaysse N., Rumeau J. L., Pradayrol L. (1995). Identification of K-ras mutations in pancreatic juice in the early diagnosis of pancreatic cancer.. Ann Intern Med.

[PDF_00589] Bishop J. M. (1991). Molecular themes in oncogenesis.. Cell.

[PDF_00590] Brentnall T. A., Chen R., Lee J. G., Kimmey M. B., Bronner M. P., Haggitt R. C., Kowdley K. V., Hecker L. M., Byrd D. R. (1995). Microsatellite instability and K-ras mutations associated with pancreatic adenocarcinoma and pancreatitis.. Cancer Res.

[PDF_00594] Clements N. C., Nelson M. A., Wymer J. A., Savage C., Aguirre M., Garewal H. (1995). Analysis of K-ras gene mutations in malignant and nonmalignant endobronchial tissue obtained by fiberoptic bronchoscopy.. Am J Respir Crit Care Med.

[PDF_00598] Der C. J., Finkel T., Cooper G. M. (1986). Biological and biochemical properties of human rasH genes mutated at codon 61.. Cell.

[PDF_00601] Emmert-Buck M. R., Bonner R. F., Smith P. D., Chuaqui R. F., Zhuang Z., Goldstein S. R., Weiss R. A., Liotta L. A. (1996). Laser capture microdissection.. Science.

[PDF_00604] Gazdar A. F. (1994). The molecular and cellular basis of human lung cancer.. Anticancer Res.

[PDF_00606] Harris C. C. (1991). Chemical and physical carcinogenesis: advances and perspectives for the 1990s.. Cancer Res.

[PDF_00608] Hollstein M., Sidransky D., Vogelstein B., Harris C. C. (1991). p53 mutations in human cancers.. Science.

[PDF_00610] Husgafvel-Pursiainen K., Hackman P., Ridanpä M., Anttila S., Karjalainen A., Partanen T., Taikina-Aho O., Heikkilä L., Vainio H. (1993). K-ras mutations in human adenocarcinoma of the lung: association with smoking and occupational exposure to asbestos.. Int J Cancer.

[PDF_00614] Keohavong P., DeMichele M. A., Melacrinos A. C., Landreneau R. J., Weyant R. J., Siegfried J. M. (1996). Detection of K-ras mutations in lung carcinomas: relationship to prognosis.. Clin Cancer Res.

[PDF_00617] Keohavong P., Zhu D., Melacrinos A. C., DeMichele M. A., Weyant R. J., Luketich J. D., Testa J. R., Fedder M., Siegfried J. M. (1997). Detection of low-fraction K-ras mutations in primary lung tumors using a sensitive method.. Int J Cancer.

[PDF_00621] Keohavong P., Zhu D., Whiteside T. L., Swalsky P., Bakker A., Elder E. M., Siegfried J. M., Srivastava S., Finkelstein S. D. (1997). Detection of infrequent and multiple K-ras mutations in human tumors and tumor-adjacent tissues.. Anal Biochem.

[PDF_00625] Khosravi-Far R., Der C. J. (1994). The Ras signal transduction pathway.. Cancer Metastasis Rev.

[PDF_00627] Li Z. H., Zheng J., Weiss L. M., Shibata D. (1994). c-k-ras and p53 mutations occur very early in adenocarcinoma of the lung.. Am J Pathol.

[PDF_00629] Marshall C. J. (1991). Tumor suppressor genes.. Cell.

[PDF_00630] Mills N. E., Fishman C. L., Rom W. N., Dubin N., Jacobson D. R. (1995). Increased prevalence of K-ras oncogene mutations in lung adenocarcinoma.. Cancer Res.

[PDF_00633] Minamoto T., Yamashita N., Ochiai A., Mai M., Sugimura T., Ronai Z., Esumi H. (1995). Mutant K-ras in apparently normal mucosa of colorectal cancer patients. Its potential as a biomarker of colorectal tumorigenesis.. Cancer.

[PDF_00640] Pretlow T. P., Barrow B. J., Ashton W. S., O'Riordan M. A., Pretlow T. G., Jurcisek J. A., Stellato T. A. (1991). Aberrant crypts: putative preneoplastic foci in human colonic mucosa.. Cancer Res.

[PDF_00643] Rodenhuis S., Slebos R. J. (1992). Clinical significance of ras oncogene activation in human lung cancer.. Cancer Res.

[PDF_00645] Rosell R., Monzó M., Pifarré A., Ariza A., Sánchez J. J., Moreno I., Maurel J., López M. P., Abad A., de Anta J. M. (1996). Molecular staging of non-small cell lung cancer according to K-ras genotypes.. Clin Cancer Res.

[PDF_00655] SLAUGHTER D. P., SOUTHWICK H. W., SMEJKAL W. (1953). Field cancerization in oral stratified squamous epithelium; clinical implications of multicentric origin.. Cancer.

[PDF_00649] Santos E., Martin-Zanca D., Reddy E. P., Pierotti M. A., Della Porta G., Barbacid M. (1984). Malignant activation of a K-ras oncogene in lung carcinoma but not in normal tissue of the same patient.. Science.

[PDF_00652] Seeburg P. H., Colby W. W., Capon D. J., Goeddel D. V., Levinson A. D. (1984). Biological properties of human c-Ha-ras1 genes mutated at codon 12.. Nature.

[PDF_00657] Sozzi G., Miozzo M., Pastorino U., Pilotti S., Donghi R., Giarola M., De Gregorio L., Manenti G., Radice P., Minoletti F. (1995). Genetic evidence for an independent origin of multiple preneoplastic and neoplastic lung lesions.. Cancer Res.

[PDF_00661] Stanley L. A. (1995). Molecular aspects of chemical carcinogenesis: the roles of oncogenes and tumour suppressor genes.. Toxicology.

[PDF_00663] Strong M. S., Incze J., Vaughan C. W. (1984). Field cancerization in the aerodigestive tract--its etiology, manifestation, and significance.. J Otolaryngol.

[PDF_00665] Sugio K., Ishida T., Yokoyama H., Inoue T., Sugimachi K., Sasazuki T. (1992). ras gene mutations as a prognostic marker in adenocarcinoma of the human lung without lymph node metastasis.. Cancer Res.

[PDF_00668] Sugio K., Kishimoto Y., Virmani A. K., Hung J. Y., Gazdar A. F. (1994). K-ras mutations are a relatively late event in the pathogenesis of lung carcinomas.. Cancer Res.

[PDF_00671] Suzuki Y., Orita M., Shiraishi M., Hayashi K., Sekiya T. (1990). Detection of ras gene mutations in human lung cancers by single-strand conformation polymorphism analysis of polymerase chain reaction products.. Oncogene.

[PDF_00675] Tada M., Ohashi M., Shiratori Y., Okudaira T., Komatsu Y., Kawabe T., Yoshida H., Machinami R., Kishi K., Omata M. (1996). Analysis of K-ras gene mutation in hyperplastic duct cells of the pancreas without pancreatic disease.. Gastroenterology.

[PDF_00683] Urban T., Ricci S., Danel C., Antoine M., Kambouchner M., Godard V., Lacave R., Bernaudin J. F. (2000). Detection of codon 12 K-ras mutations in non-neoplastic mucosa from bronchial carina in patients with lung adenocarcinomas.. Br J Cancer.

[PDF_00679] Urban T., Ricci S., Lacave R., Antoine M., Kambouchner M., Capron F., Bernaudin J. F. (1996). Codon 12 Ki-ras mutation in non-small-cell lung cancer: comparative evaluation in tumoural and non-tumoural lung.. Br J Cancer.

[PDF_00690] Westra W. H., Baas I. O., Hruban R. H., Askin F. B., Wilson K., Offerhaus G. J., Slebos R. J. (1996). K-ras oncogene activation in atypical alveolar hyperplasias of the human lung.. Cancer Res.

[PDF_00687] Westra W. H., Slebos R. J., Offerhaus G. J., Goodman S. N., Evers S. G., Kensler T. W., Askin F. B., Rodenhuis S., Hruban R. H. (1993). K-ras oncogene activation in lung adenocarcinomas from former smokers. Evidence that K-ras mutations are an early and irreversible event in the development of adenocarcinoma of the lung.. Cancer.

[PDF_00693] Wilentz R. E., Chung C. H., Sturm P. D., Musler A., Sohn T. A., Offerhaus G. J., Yeo C. J., Hruban R. H., Slebos R. J. (1998). K-ras mutations in the duodenal fluid of patients with pancreatic carcinoma.. Cancer.

[PDF_00696] Yakubovskaya M. S., Spiegelman V., Luo F. C., Malaev S., Salnev A., Zborovskaya I., Gasparyan A., Polotsky B., Machaladze Z., Trachtenberg A. C. (1995). High frequency of K-ras mutations in normal appearing lung tissues and sputum of patients with lung cancer.. Int J Cancer.

[PDF_00700] Yanagisawa A., Ohtake K., Ohashi K., Hori M., Kitagawa T., Sugano H., Kato Y. (1993). Frequent c-Ki-ras oncogene activation in mucous cell hyperplasias of pancreas suffering from chronic inflammation.. Cancer Res.

[PDF_00703] Zhu D., Keohavong P., Finkelstein S. D., Swalsky P., Bakker A., Weissfeld J., Srivastava S., Whiteside T. L. (1997). K-ras gene mutations in normal colorectal tissues from K-ras mutation-positive colorectal cancer patients.. Cancer Res.

